# Kyasanur Forest Disease vaccination coverage and its perceived barriers in Goa, India—A mixed methods operational research

**DOI:** 10.1371/journal.pone.0226141

**Published:** 2019-12-31

**Authors:** Annet Oliveira, Kalaiselvi Selvaraj, Jaya Prasad Tripathy, Utkarsh Betodkar, Jagadish Cacodcar, Abhijit Wadkar

**Affiliations:** 1 Integrated Disease Surveillance Programme, Directorate of Health Services, Panaji, Goa, India; 2 All India Institute of Medical Sciences, Nagpur, India; 3 International Union Against Tuberculosis and Lung Disease, Paris, France; 4 State Surveillance Officer, Directorate of Health Services, Panaji, Goa, India; 5 Department of Preventive and Social Medicine, Goa Medical College, Bambolim, Goa, India; 6 Medical Officer, Directorate of Health Services, Panaji, Goa, India; Australian Red Cross Blood Service, AUSTRALIA

## Abstract

**Background:**

Kyasanur Forest Disease (KFD) is a highly infectious viral illness transmitted by infected ticks through contact with monkeys and other forest animals. Till date there is no definite treatment available for KFD. Hence, vaccination is considered to be an important public health intervention to control KFD. This study aimed at estimating the vaccination coverage for primary and booster doses of KFD vaccine and exploring the perceived barriers to vaccination in the affected villages of Goa, India during 2015–18.

**Methodology & principal findings:**

In this explanatory mixed methods study, vaccine coverage was estimated bydata obtained from the KFD vaccination registers maintained at the health centers catering to the KFD affected villages. To understand the barriers to vaccination,key informant interviews were conducted among implementing health officers, medical officers and nurses involved in vaccination. Perceptions of vaccinees and community members were studied through in-depth interviews and focus group discussions.

Out of the 35,500 targeted population (6–65 years)for KFD vaccination, 32% received one dose and 13.2% received two doses. The coverage for first booster and annual booster was 4.9% and 0.5% respectively. The drop out from first to second and third doses was 57% and 85% respectively. 69% of doses were delivered during community outreach programmes and remaining at health facilities. Inadequate vaccine stock, inappropriate timing of vaccination campaign, lack of awareness and misconceptions related to indications of vaccines, travel distance for follow up doses given at community health centre and pain due to injection were perceived as reasons for poor vaccination coverage.

**Conclusions:**

KFD vaccination coverage was poor in the villages affected by KFD in Goa. Both left-out and drop-out phenomena were observed in KFD vaccination. Vaccine implementation plan has to consider suitable time for the local people, maintain adequate vaccine stock and encourage community-based vaccination campaigns instead of facility-based to achieve optimal vaccine coverage.

## Introduction

Kyasanur Forest Disease (KFD) is one of the emerging zoonotic viral infections transmitted by infected ticks. Since 1957, Shimoga and adjoining districts of Karnataka were regularly reporting cases in India. In recent years, adjoining border districts of States such as Goa, Maharashtra also are reporting cases of KFD[1,2]. Annually, around 400–500 cases are reported in India [3]. In Goa, the KFD outbreak was first reported in Pali village of Sattaritaluka in 2015; since then there has been a spread to other villages [1].

KFD presents with features of viral haemorrhagic fever and remains an important differential diagnosis in the evaluation of tropical fever. KFD is a highly infectious viral disease which needs high level of biosafety (level 3) monitoring for testing[4]. For the same reason testing facilities are available only at regional laboratories. Apart from febrile features, KFD also results in neurological dysfunction and death in some cases. The case fatality due to KFD was reported to be 2–10%[5]. Till date, there is no definite treatment available apart from supportive therapy. Due to its nature of zoonotic spread and enhanced transmission through bio diversity related issues which often does not come under the control of the health sector, implementing public health interventions is challenging.

Vaccination is considered to be an important public health intervention to control KFD. There is limited availability of information on KFD vaccine for human use in India. Indigenously manufactured vaccine is made available in the endemic districts through the Institute of Animal Health and Veterinary Biologicals in Bangalore, Karnataka [5]. In the KFD endemic districts of Karnataka, vaccination remains a key control strategy. It is considered to be a promising cost effective strategy in the control of KFD in emerging districts also. Till date, there is no proven human to human transmission of the virus. Hence, there is no concept of herd immunity in the prevention of KFD. So, to ensure protection from the disease all vulnerable population should be targeted for vaccination. For optimal vaccination response, the following vaccination schedule is followed: two dose of vaccine over one month interval, first booster dose after 6–9 months after primary vaccination, thereafter annual booster doses for 5 consecutive years after the last confirmed case in that area [6].

Vaccine efficacy of KFD varies with the number of doses received. Person receiving only one dose is as susceptibleto KFD as those unvaccinated. Person receiving two doses and one booster dose have 62% and 83% lesser incidence of the disease respectively. Previous published evidences have documented poor vaccination coverage for any dose of KFD vaccine [6,7]. This indicates that though there is an effective vaccine available, the implementation of the vaccination strategy has not been successful.

Following the outbreak of KFD in Goa, vaccination strategy was implemented in 2015. However, anecdotal evidence from the programme staff indicates poor coverage of the vaccine among the users, especially for the second and the booster doses. This can occur both due to supply and demand side factors. Achieving optimal vaccination coverage in the affected villages needs an in-depth understanding of the barriers against uptake of KFD vaccination. Thus, we carried out a mixed methods study with the following objectives, i) estimate the vaccination coverage by a desk review of vaccination campaign reports available at the health centresserving the KFD affected villages in Goa followed by a ii) qualitative enquiry to explore barriers to the uptake of KFD vaccination and suggestions from a users’ and providers’ perspective.

## Methods

### Study design

This was an explanatory mixed-methods study design with a cross-sectional analytical quantitative component using secondary data (reports of vaccination campaign), followed by a descriptive qualitative component[8].

### Setting

#### General setting

Goa is a state located along the coastal region of Western India. The border areas of Goa have many dense forest regions which are endemic for KFD due to the presence of ticks in those regions.

#### Specific setting

In Goa, the KFD outbreak started in Pali village of SattariTaluk, North Goa in 2015. In the same year a total of 36 cases occurred and majority of the cases were distributed within 5 kilometres of the index case. Over the years there has been a spread of the disease to other villages. The index village and surrounding villages are located near the western region with dense forests nearby. Most natives residing there are poachers or cashew harvesters and are regular forest goers. Considering the rise in cases of KFD, the state health mission of Goaannounced mandatory reporting of probable cases of KFD to the IDSP unit of Goa from all Peripheral Health Institutes. The health department also introduced KFD vaccination in the index village and surrounding villages from where cases have occurred. As per the schedule each person has to receive two doses (primary doses) one month apart and annual booster (booster doses) afterwards for five years since the occurrence of the last case in the village.

The focal immunization strategy involved annual rounds of vaccination. These campaigns were conducted during the months of August-November in the areas that reported KFD activity (defined as laboratory evidence of confirmed case/s in monkeys/humans or infected ticks) in the previous transmission seasons and surrounding villages within a radius of 5 kilometers. [9] In 2015, vaccination for KFD was given at Community Health Centre (CHC), Valpoi only which is a secondary level health facility catering a population of around 43130 in the KFD affected region. However, vaccine coverage remained poor. In order to increase the coverage of KFD vaccine, community based vaccination campaigns in the affected villages started in March 2016. Mass vaccination campaigns, out-reach sessions were held in the community in the affected villages by the village level health workers. The staffs carrying out immunization sessions at the village level are supposed to submit periodically the aggregate report of the eligible population, number vaccinated with KFD and number of cases of KFD in the village. They are also supposed to maintain a line list of people vaccinated with age, sex, number of doses received and data of vaccination. These aggregate reports and line lists are regularly submitted to the State Surveillance Unit of Integrated Disease Surveillance Programme in a structured format.

### Study population

#### For the Quantitative phase

To assess the vaccination coverage, aggregate number of people in the age group of 6–65 years (eligible for vaccination) living in villages where vaccination campaigns were conducted was considered as the study population. Out of this eligible population in the affected villages, number of persons vaccinated with 1^st^ dose, 2^nd^ dose and the subsequent booster doses were analyzed from the available vaccination records.

#### For the qualitative phase

Key Informant Interviews (KIIs) were conducted with State Surveillance Officer, Health Officers, Medical Officers and ANMs (Auxiliary Nurse Midwives) involved in vaccination campaigns at their work place. Adults from the affected villages where vaccination campaign were held constituted the study population for focus group discussions (FGDs).

### Data variables, sources of data and data collection

#### Phase1: Quantitative data collection

The data on vaccination coverage were obtained from the KFD vaccination registers maintained at the public health facilities (Primary Health Centres/PHCs) attached to the affected villages. The target population (6–65 years) was obtained by contacting the ANMs working in the affected villages. For those affected villages where population could not be obtained, an estimated population based on census 2011 was used[10]. The vaccination registers had names of the patients vaccinated,area residing, place of vaccination along with the date of vaccination. Village wise aggregate figures of vaccinations received (first dose, second dose and booster dose) were obtained from these registers. Using the village administrative map of Goa, cases per 1000 population is depicted in the form ofadensity map using software named Quantum Geographic InformaitonSystem (QGIS). Vaccination coverage is also depicted as density map through Quantum GIS.

#### Phase 2: Qualitative data collection

Two categories of key informants were interviewed. The first category included the officer in-charges who were involved in the planning of the vaccination program. The second category was the health personnel involved in implementing the vaccination drive i.e the ANMs and the nursing staff. Using an interview guide (Appendix A), the Principal Investigator (AO who is a lady Medical Doctor) who is trained in qualitative research conducted all the 13 KIIs to explore the challenges faced during the implementation of KFD outbreak vaccination campaigns and the reasons for poor coverage. One of the staff who was interviewed said that she was new and had joined three months back with no field experience in KFD vaccination. She was excluded and it was decided to interview only those who have worked for at least a year in the affected villages. Saturation of findings determined the sample size of KIIs.

Two FGDs were conducted in two randomly selected affected villages to explore their views on KFD vaccination and reasons for poor uptake of the vaccine (Appendix B). The 1^st^FGD was conducted at Compordem village at the sub-centre with 8 participants making sure that the group is homogeneous with respect to the receipt of vaccination(Four were vaccinated and four unvaccinated) and also with respect to gender (3 males and 5 females). The 2^nd^ FGD was conducted at the Anganwadi of village Pali with 8 female participants (2 elderly females and the rest between the age group of 20–40). Two participants were working as government servants and the rest were homemakers. The PI who was a female doctor conductedboth the FGDs at a time suitable to the participants in the community. The FGD participants were informed of the purpose of the study and those willing to participate were invited. Both the FGDs were conducted in the local language in which the PI was also comfortable with. The FGDs went on for around 30–40 minutes. At the end of the interviews/FGDs the summary was read back to the participants for validation.

### Ethical issues

Ethics approval was obtained from the State Ethics Committee, Directorate of Health Services, Goa (24–166) and the Ethics Advisory Group of the International Union Against Tuberculosis and Lung Disease, Paris, France(93/18). Written informed consent was obtained from the participants before conducting the FGDs and KIIs.

### Analysis and statistics

#### Quantitative

Data were entered in Microsoft Excel spreadsheet and analyzed using Epidata analysis (V2.2.2.183). Vaccine coverage and drop out for the subsequent doses were expressed as percentages. Operational definitions used in estimating the vaccination coverage’s are given in Table 1 below. The factors associated with vaccine coverage for the first dose was explored using adjusted linear regression technique. The following factors were explored as explanatory variables: i) Eligible population, ii) Number of confirmed KFD cases in the villages iii) Distance to the nearest CHC, iv) Time taken to reach the nearest CHC. The outcome and explanatory variables were aggregate level information at the village level (ecological data). Correlation coefficient and beta co-efficient of linear regression are given for each factor. Using the village administrative map of Goa, cumulative cases per 1000 population and vaccination coverage for at least one dose is depicted in the form of density map through QGIS software.

**Table 1:** Operational definitions used in estimating vaccine coverage for different doses.

**Vaccine Coverage for 1**^**st**^ **dose in a village**	(Total number aged 6–65 years who have
	received 1^st^ dose in the village)/(Total number of
	6–65 year old in the village)
**Overall vaccination coverage for 1**^**st**^ **dose**	(Total number aged 6–65 years who have
	received 1^st^ dose in the village)/ (Total population
	aged 6–65 years in all KFD affected villages)
**Overall vaccination coverage for 2**^**nd**^**dose**	(Total number aged 6–65 years who have
	received 2^nd^dose in the village)/ (Total population
	aged 6–65 years in all KFD affected villages)
**Overall vaccination coverage for 3**^**rd**^**dose**	(Total number aged 6–65 years who have
	received 3^rd^dose in the village)/ (Total population
	aged 6–65 years in all KFD affected villages)

#### Qualitative

KIIs/FGDS were transcribed by the PI (AO) on the same day. Transcripts were read several times by the PI and another co-investigator (KS). Codes were generated by iterative inductive coding approach by two independent trained investigators in the study (AO& KS). Similar codes were combined to make categories and themes. Any discrepancy was resolved through discussion among the authors (AO, KS and JPT). Results have been summarized in the form of a thematic framework. We have adhered to the Consolidated Criteria for Reporting Qualitative Research (COREQ) to report the qualitative findings [11].

## Results

### Vaccine coverage

A total of 40 villages had reported at least one confirmed case of KFD during 2015–2018. Vaccination campaigns started in April 2015 at Pali village and from March 2016 onwards in other villages. Overall, a total of 15 villages had vaccination coverage less than 25%, 17 villages had coverage ranging from 25–50%, vaccine coverage of 50–75% was seen in 04 villages and another 04 villages showed high vaccine coverage >75%.

**Fig 1:**
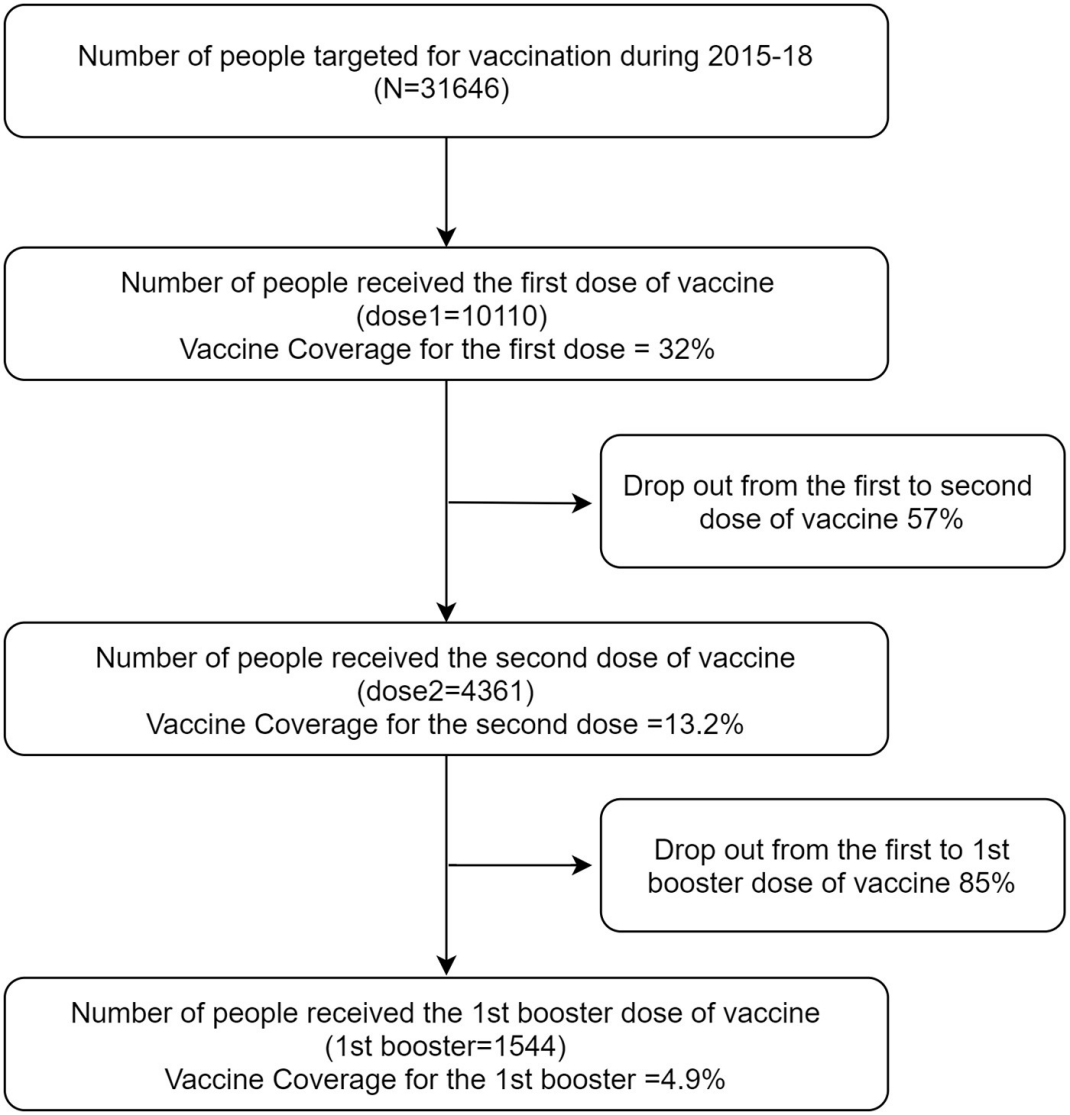
Flow diagram depicting vaccination coverage for KFD and vaccine dropout rates in affected villages of Goa, 2015–18.

Of the 16141 KFD including126 second booster dosevaccinations in the affected villages, 31% were delivered in facilities such as the PHCs and CHCs and the remaining 69% doses were in the community outreach programmes. Overall, 32% of the eligible population received at least one dose of KFD vaccine. The drop out from first to second and third doses was 57% and 85% respectively(Fig 1).

**Fig 2:**
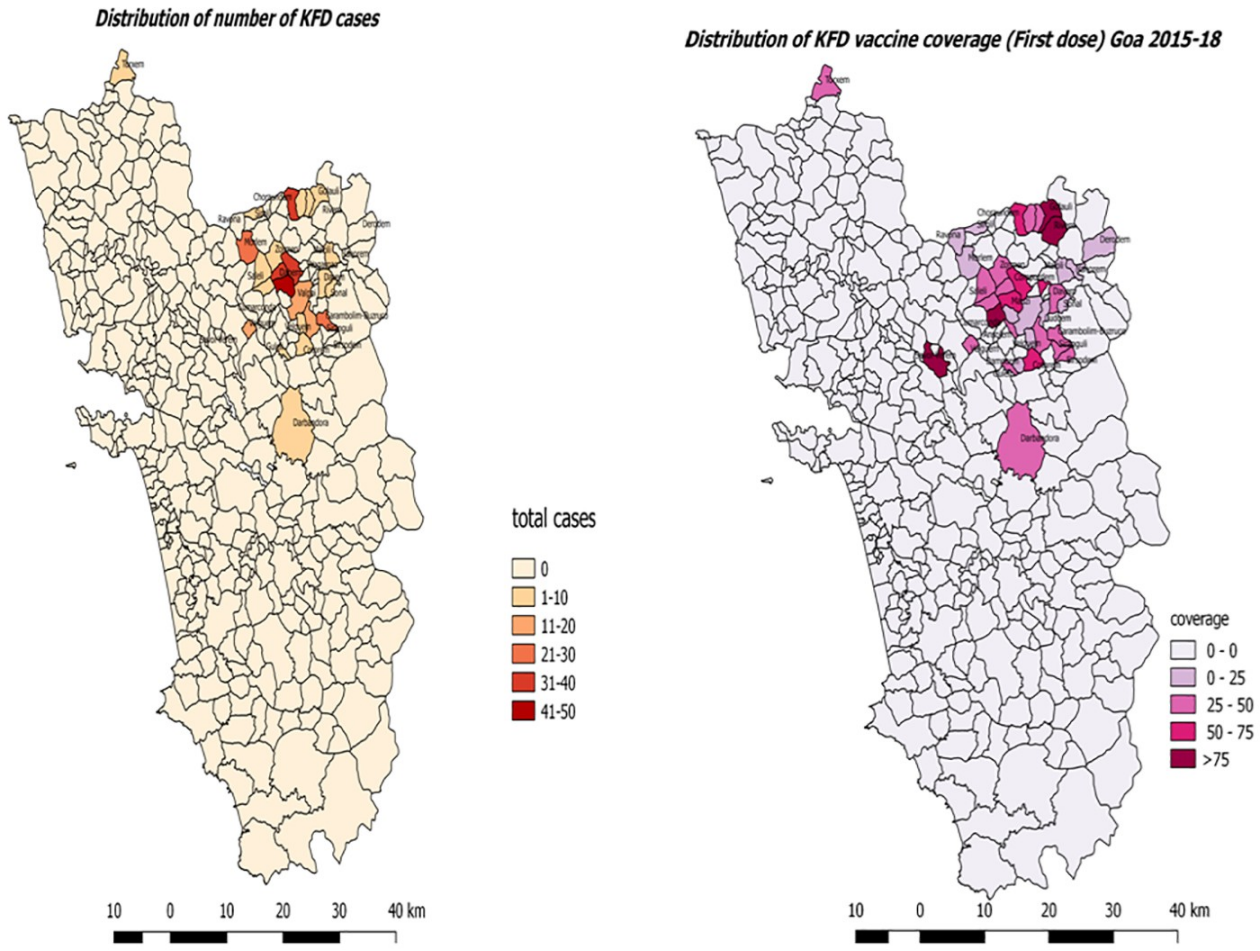
Village-wise KFD cases and vaccination coverage, Goa, India, 2015–2018[maps created for this present study using OGIS (S2 File).

Among the top 15 villages with more number of cumulative cases, except three all other villages had at least 35% vaccination coverage. (Fig 2). Though number of target population to be vaccinated showed a negative correlation with first dose vaccine coverage the findings were not statistically significant. Similarly, other factors such as distance to the nearest CHC and time taken to reach the nearest CHC were also not associated with vaccination coverage (Table 2). (S1 File).

**Table 2:** Factors associated with KFD vaccination coverage for first dose using unadjusted linear regression analysis.

Factor	Correlation	Beta coefficient	P value
**Cumulative number of confirmed KFD cases (2015–18)**	0.17	0.34	0.3
**Target population for vaccination in a village**	-0.07	-0.003	0.4
**Distance to the nearest CHC**	0.14	0.63	0.8
**Time taken to reach the nearest CHC**	0.13	-0.24	0.9

Dependent Variable: Vaccination coverage for first dose KFD; Independent variables: Cumulative number of KFD cases, Number of targeted population, Distance and time to travel. KFD: Kyasanur Forest Disease; CHC: Community Health Centre

### Qualitative findings

Facilitating factors for vaccination: Medical officers and implementing health authorities for the vaccination programme mentioned the following as facilitating factors for KFD vaccination: i) Due to the poor vaccination coverage in facility based vaccine delivery, a decision was made to shift the vaccine camps to sub-centres on fixed days with prior dissemination of the message through various modes of communication, ii) To implement safe vaccine delivery, all health care professionals were trained in cold chain maintenance, micro-planning and aseptic precaution methods. iii) Vaccination card was issued to all vaccinees with column for writing the date for subsequent dose, iv) Since this was a new vaccine, to increase the acceptance among community members, vaccination campaigns were started in the presence of Panchayat Raj members, Medical officers and Health officers of the nearby PHCs v) There was an official order circulated among employees working in forest and animal husbandry department (who are more vulnerable to have contact with animals) to get vaccinated against KFDin specific health facilities (PHCS/CHCs), and vi) To raise awareness regarding vaccination, opportunities such as household visits for distributing Dimethyl phthalate topical applications were utilized.

**Fig 3:**
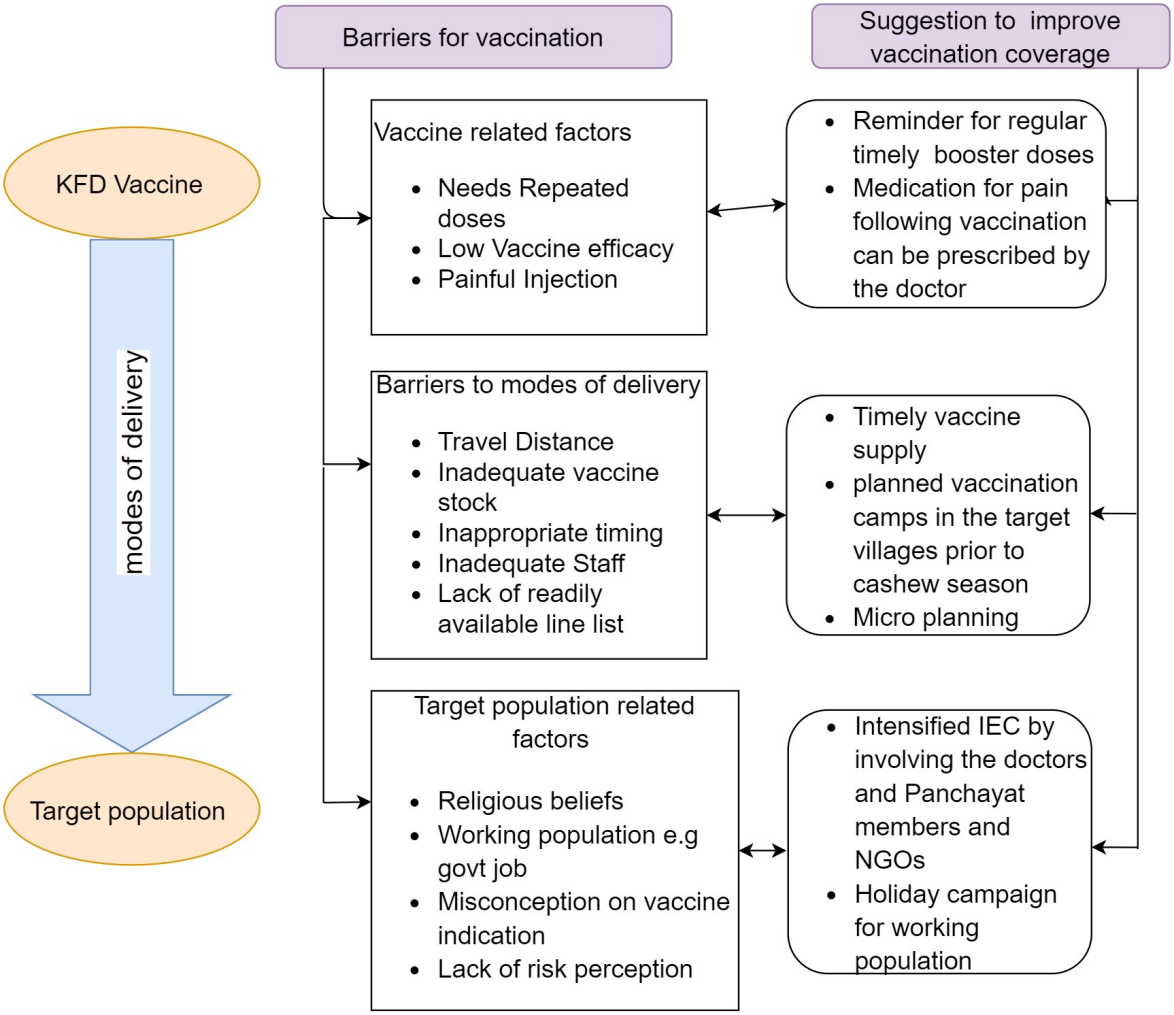
Barriers for vaccination and suggestions to improve coverage.

The barriers and suggested solutions are summarized in Fig 3.

#### Perceived barriers

Barriers related to poor vaccination coverage were analyzed from the transcripts of KIIs and FGDs among community members. Health care providers perceived vaccine stock related issues and distance to be the major barriers to adequate vaccine coverage. The community members perceived vaccine related reactions, inappropriate timing of vaccination campaign during cashew harvest season and lack of awareness as the key reasons for non-vaccination. Details of barriers and supporting quotes are summarized in (Table 3).

**Table 3:** Perceived barriers to vaccination coverage in the KFD affected villages, Goa, India.

Category	Codes	Quotes
**Vaccine related barriers**	Inadequate vaccine stock Poor vaccine efficacy Vaccine reactions & contra indications Repeated doses	“Vaccine stock was not available in the beginning of the outbreak”**(Doctor)** “Later in 2018 when there were more cases vaccine stock was less”**(Nursing staff)** “I had gone to the hospital for subsequent dose but that time it was informed to me that vaccine in not in stock so it did not go again” **(Elderly females from the affected village)** “Some cases have occurred even after Vaccination”**(Doctor)** “When the injection was given I felt it was like a shock and Injection was so painful”**(Vaccinee)**“Pain remains even for 1 to 2 days”**(Vaccinee)** “Some of the people who took the vaccine went and told the others that vaccine is painful and hence some did not come to take the vaccine”**(ANM)** “Each person prior to vaccination has to undergo detailed evaluation to rule out several contra-indications such as pregnancy, IHD, Seizure disorder, ALD” **(Doctor)** “People took the first 2 doses but some did not come for 3^rd^ dose”**(Health care provider)**
**Barriers in delivering the vaccine to the target population**	The distance from health facility to community Inappropriate timing	“There are many far off villages like almost border area and far to reach areas”**(Doctor)** “Interior villages so transport is difficult” (**ANM)** “Because people have to travel to CHC for follow up doses they do not go” **(Community member)** “People are busy with cashew harvest in the peak season of KFD (Feb–March)” **(Health care provider)**
**Barriers at the level target population**	Lack of awareness Religious belief Misconception Occupation Lack of risk perception	“Most of the people in the beginning did not know about the importance of vaccine.” “I was not aware of the fact that yearly booster doses are needed” **(elderly female User)** “There was a religious festival that happened, so people thought that the disease was due to things that did not went proper duringthe festival like outsiders who attended the festival” **(Anganwadi worker from the village)** “I thought the vaccine was to be taken by people who develop the symptoms of KFD.**(student from the village)** “I was not having any symptoms of KFD hence he did not take the vaccine”**(Male 50years)** “Difficult to get people for vaccination during cashew harvest season” **(Community member**) “People working as Govt servants might have missed the dose…” Disease was there that time people took the vaccine. Now since there are no cases people do not feel it as important anymore (**Community member**) People say that they do not go to the forest. So why to take the vaccine? (**ANM**)

Suggested solutions: Both provider and the community members felt that giving all doses at sub-centre itself will improve the vaccination coverage for follow-up doses. Community members said that if the sub-centre vaccination campaigns are scheduled on holidays, employees and students who go out during the weekdays also would get the vaccine.

“See if vaccination time and date can be fixed on a day where most of the villagers including the working population will be available like on Sundays”

(Community member)

The health officer had opined that the planning of vaccination campaign before December would increase the vaccination coverage because people generally do not go for cashew harvesting during that period and that is the period when the nymph activity of tick population and transmission is low.

“Plan vaccination campaigns in the affected areas prior to the season when cases peak. This silent period can be used for maximum coverage”

(Health Officer)

Health care providers also said that involvement from the community such as the presence of PanchayatRajmembers during the campaign, training of Anganwadi workers to disseminate the information and intensive Information Education Communication could increase the coverage.

“To vaccinate the people on the same day where a major awareness campaign can be held involving the doctors and panchayat members because when doctors and local member is present the turnover is more”

(KII-ANM)

One of the implementing officers also suggested daily reporting of vaccination which will update the programme authorities and aid in intensive monitoring.

## Discussion

This is the first mixed methods paper looking at KFD vaccination coverage and drop outs and the reasons for poor vaccine coverage. The current study analyzing routine reporting of KFD vaccination campaign revealed poor vaccination coverage for 1^st^ dose i.e. 32%. The coverage for subsequent doses is further low: 13.2% for second dose and 4.9% for the first booster dose. The other study from Karnataka which assessed vaccine effectiveness in community based settings also reported similar figures for first (23.4%) and second doses (15.4%) [7]. Contrary to this, vaccine coverage reported from five endemic districts of Karnataka during 2005–10 was higher: around 48% for any dose and 36% for two doses and a booster dose [6]. The probable reason for better vaccine uptake in this study could be due to increased awareness about the disease during the initial years of outbreak (2005–10) in the study districts.

In the present study, the coverage for first booster was only 4.9% which was low and only 0.5% for second booster. High drop-out of booster doses is a well reported phenomena in routine immunization for vaccines where multiple doses are required [12]. Same is the case for KFD vaccine as well [6]. This poor KFD vaccination coverage could be due to lack of awareness as reflected by community members during the FGDs and lack of perceived susceptibility due to declining number of cases and villages affected by KFD. Need to go to the CHC for vaccination was perceived by the community members as well as the providers as a reason for not getting vaccinated due to access barriers.

During the FGDs and interviews, people had expressed their perceived need for vaccination in the villages where the cases were more. The least vaccination coverage in 2018 could be explained by inadequate vaccine stock. This emerged from multiple health care providers’ interviews as well. This requires adequate measures to ensure vaccine stock and meticulous micro-planning.

The study had the following strengths. This is the first study reporting vaccine coverage at a village level by analyzing routine programmatic reports. A mixed methods study design allowed us to explain the reasons for poor vaccine uptake from both health care providers’ and community perspectives.

There were few limitations in this study. This study estimates the vaccine coverage in villages where there is occurrence of at least one case in four years. However, there could be villages which had vaccination campaigns based on the policy of being located within 5 kilometers of the affected village or reported monkey deaths from the forest department. These villages were not included in the overall estimation of vaccine coverage; although they were limited in number i.e. 6 villages only. We feel that inclusion of those villages might have lowered the vaccine uptake as we expect lesser anticipated response in villages where no case of KFD has been detected. Since the vaccination registers reported aggregate figures, association of individual factors such as education, income, occupation associated with poor vaccination coverage could not be studied.

The study findings have the following four programmatic implications. i) Logistic issues such as vaccination stock should be made available in co-ordination with the district immunization office ii) The distance and the need to go to the nearest CHC was an important barrier for second and booster doses. Hence, community based vaccination campaign should be expanded for second dose and booster doses of KFD also, iii) Misconceptions related to the vaccine and its side effects, vulnerable population for KFD and the schedule for vaccination should be clarified with the involvement of local village level grass root workers such as Anganwadiworkers.iv) To achieve optimal vaccination coverage, vaccination campaign should be planned preferably before November so that majority of the vulnerable population especially those who are involved in cashew harvest get vaccinated as it does not coincide with the harvesting season. Since it takes around two months to develop sufficient level of antibodies, conducting vaccination campaigns before November would prime the susceptible population with a better immune state at the time of peak transmission i.e. January-March.

## Conclusion

KFD vaccination coverage was poor in the KFD affected villages of Goa. Considering the cashew harvesting season, vaccination campaigns should be planned around November in order to maximize coverage. This will also ensure that the vulnerable populations are immune before the start of the peak transmission season. Maintenance of adequate vaccine stock and raising community awareness about the disease and the role of vaccine is needed. We should also encourage community-based vaccination campaigns over facility-based to achieve optimal vaccine coverage.

## Supporting information

S1 File Vaccination coverage and studied factors affecting vaccination coverage. (XLSX)

S2 File Yearwise and areawise KFD cases in the state of Goa. (XLSX)

## Appendix A

### Topic guide for KII

Interviewer: Good morning. Thanks very much for consenting to be a part of this study.

Rapport building: As we knew that since 2015, Goa is facing a regular outbreak of Kyasanur Forest Disease. Since there is no definite treatment is available we are dependent supportive theraphy and public health interventions to reduce morbidity and mortality.

One of the promising public health measure suggested from the previous endemic states was vaccinating the high risk individuals residing in the affected community.

1. What is your opinion on the vaccination strategy?
2. As an implementing programmeco-ordinator/ implementing health officers/ implementing health worker in the field what were the challenges did you encounter?
  probe: what was the vaccination coverage.
3. in your opinion, what were the major reasons for poor vaccination coverage?
4. what were the strategies adopted to improve the vaccination coverage?
5. what did you plan for future vaccination campaign to achieve optimal vaccination coverage?
6. From your experience in the field, Can you share some solutions for the health system to take it up while they plan for these kind of vaccination campaign?

Thanks for your valuable time shared with us. Do you want to share with us anything more?

## Appendix B

### Topic guide for Focus Group Discussion

Interviewer: Good morning all of you. Thanks very much for consenting to be a part of this study.

Rapport building: As we knew that since 2015, Goa is facing a regular outbreak of Kyasanur Forest Disease. Since there is no definite treatment is available we are dependent supportive theraphy and public health interventions to reduce morbidity and mortality.

One of the promising public health measure suggested from the previous endemic states was vaccinating the high risk individuals residing in the affected community.

Since this village is being regularly affected due to KFD we feel as a part of this community you all can provide us valuable information related to this disease. As I ask some queries from you one by one can answer for that. When one person is answering do not interfere or stop them. Wait for your turn and everybody have equal rights to share their views. You need not worry whether your views are right or wrong. We just want to capture what you feel about this particular issue.

1. What is your experience on this KFD disease?
2. How did you come to know regarding the vaccination?
3. Were there difficulties faced by the community members at the time of KFD vaccination? If yes then what were they probe: in accessing the vaccine?
4. In your opinion, what were the major reasons for not accepting the vaccine by the community members?
5. As there are members in this FGD who have already been vaccinated, what were the motivating reasons to go for the vaccination?
6. We came to know for the second dose and booster doses very less number of people got vaccinated. What was the reason people did not turn for the second dose or booster doses?
7. From your experience, do you want to give some suggestions to the health system to take it up while they plan for these kinds of vaccination campaign?

Thanks for your valuable time shared with us. Do you want to share with us anything more?
