# Supplementary material for: Kyasanur Forest Disease vaccination coverage and its perceived barriers in Goa, India—A mixed methods operational research
Source: PLoS One. 2019 Dec 31;14(12):e0226141. doi: 10.1371/journal.pone.0226141 (PMC6938374; doi:10.1371/journal.pone.0226141)
Supplement: Attachment — Submitted filename: Response to all reviwers.docx [file pone.0226141.s003.docx]

Reviewer 1

1. The error in the following sentence “pain due to were injection” in the Abstract

Reply: Is changed to “pain due to injection were”

2. Introduction: Regarding the sentence “Indigenously manufactured vaccine with good efficacy is made available in the endemic”

Reply: we have omitted the words good efficacy and added a line No. 87-88 “There is limited availability of information on KFD vaccine for human use in India.”

3. Why village populations were not available for few villages?

Reply: The population was used as a denominator to calculate the coverage for the specific year when cases occurred in the village. Few villages the ANMs were new who had only the recent population of 2019 only those old ANMs who werte transferred or left the job who could not be contact the an estimated population based on census 2011 was used.

4. Sentence “The PI (AO- lady Medical Doctor) who is trained in qualitative research” previously mentioned in the methods section.

Reply: line 222 in the older version is cancel to avoid repetition as per your comment.

5. “Of the 16141 KFD vaccinations” line No 261. Fig 1 shows: 6015

Reply: The reason for the difference is Fig 1 has shown up to 1^st^ booster dose. 126 people had received the 2^nd^ yearly booster i.e. a 4^th^ dose. We have made a mention of this in line no 261.

6. Suggestion for pain and low efficacy in Fig 3.

Reply: Corrected Fig is uploaded

Reviewer 2

1. A more complete description of the methodology and the study population would significantly improve this manuscript.

Reply: Thank you for the comment. We agree with your point. In the revised manuscript we have elaborated on the context which the immunization campaign was held, data available within the programme (IDSP unit), villages covered and study population. We hope the revised manuscript will give a better understanding and coherence for the reader. Line Nos. 132-143 and 152-164

2. The data are not clearly presented in either text or table format.

Reply: Since the manuscript is based on the observations of a mixed methods evaluation which comprises of findings from qualitative as well as quantitative it might have generated the impression of ambiguity. However, based on the comments we have tried to organize our tables by removing redundant information and adding additional details to the given tables.

Since we felt the thematic GIS map will give better visual information related to the coverage and barriers we have retained the same. To avoid redundancy we have removed Table 2. In Table 3, we have added in the foot note what is response (outcome) variable and what is exposure variable for more clarity.

We have tried to consolidate Table 4 by removing repetitive quotes and have kept one verbatim quote for each code. We hope that with the current revised version, a better logical sequence and clarity in presentation is achieved.

3. The conclusions are generally supported by the presented data, but are very much descriptive.

Reply: Thank you for the comment. We have limited our conclusion to the findings in the study and have given relevant and practical recommendations based on the study findings. We have given more specific recommendations now.

4. The authors need to proof this manuscript very carefully. Many typos and grammatical errors throughout.

Reply: Agreed. The whole manuscript has been subjected to review by language experts.

5. In this study, the authors queried the local population regarding their vaccine coverage and why they did not receive the complete vaccine series, if that was the case.

Reply: Yes we did involve the community members in the Focus Group Discussions (FGDs). In the 2 FGDs conducted at 2 different affected villages the participants were local population who were asked about the difficulties, beliefs or misconceptions regarding KFD vaccine.

As per our analysis, the coverage for the first and second dose of vaccine is 32%, 13.2% respectively. The coverage for booster dose is still low to the extent of 4.9%. Since the vaccination campaign report does not have documented reason the individual person the reason for not vaccination or drop out from first to second or for further subsequent doses we cannot state the reason quantitatively.

However, to understand we have explored through qualitative interviews. Results of these interviews had shown the issues such as inappropriate timing, out of stock, misconceptions and distance to be deterrent factors against vaccination. These reasons along with suggestions we depicted in figure 3.

6. However, many of the details are lacking and the data are not presented in a way that is either complete or especially clear. The authors need to be very clear on how they collected the data, the number of people queried and more specific demographics of the people queried (i.e. more specific age range, gender, occupation, etc).

Reply: We have described the population interviewed in detail, but to maintain confidentiality we cannot specify more details as the group interviewed is small (13 participants for KII, 8 participants od 1^st^ FGD and 8 participants in the 2^nd^ FGD] Line Nos. 214-221.

In the quantitative component, since the programme report does not have mandate to collect information such as occupation we were not able to present those.

However, since qualitative interviews were conducted prospectively after planning of the study we had data of all the people who participated in the interview. We have incorporated these observations in the revised manuscript

7. For example, the information in Table 2 and the following paragraph should be combined to provide more complete information in a single table.

Reply: Agreed. For better clarity and avoid redundancy we have removed table 2 and incorporated the information in the text. Line Nos. 274-275

8. I don’t understand the point of Table 3 since correlation values are provided, but not an indication of what is actually being correlated.

Reply: Thank you for the comment. For better clarity we have mentioned the response variable and exposure variables clearly in the foot note and the same we have incorporated in the statistical analysis plan as well.

9. The information in Table 4 should also be consolidated.

Reply: Since the table is to present the qualitative findings we tried to justify each codes with appropriate verbatim quotes. However, as per the suggestion we have tried to consolidate the table by removing the redundant quotes.

10. Specific comments:

The authors need to carefully proof this document.  There are a number of typographical and factual errors that need to be addressed throughout the document.

Reply: We have gone through the manuscript to correct the typographical errors.

11. Please provide line and page numbers.

Reply: Page No and line No has been provided.

12. The reference to “AO” as a “lady Medical Doctor” needs to be clarified.  AO is either a doctor/physician (gender is not relevant) or maybe a gynecologist/obstetrician.

Reply: Lady was with reference to gender. The mention of this was done in the FGD because the 2^nd^ FGD all the participants were females so we thought it would be relevant to make a mention of the gender of the Principal investigator who conducted the interview. The PI was a female doctor.

Reviewer 3

Introduction

1. Consider providing reference/s to the sentences about KFD outbreak in Pali, and KFDV is class III biosafety pathogen.

Reply: Agreed. Thanks for the comments. As per the suggestion we have added the reference to these statements.

**Reply: The following references are added:**

KFD outbreak in Pali: Ref 1

Murhekar MV, Kasabi GS, Mehendale SM, Mourya DT, Yadav PD, Tandale BV.On the transmission pattern of Kyasanur Forest disease (KFD) in India. Infect Dis Poverty. 2015 Aug 19;4:37. doi: 10.1186/s40249-015-0066-9. PubMed PMID: 26286631;PubMed Central PMCID: PMC4545326.

Ref 4: PKFDV is class III biosafety pathogen : Pragya D. Yadav a, Anita M. Shete a, Deepak Y. Patil a, V.K. Sandhya b, K.S. Prakash c, Rajesh Surgihalli b, Devendra T. Mourya. Outbreak of Kyasanur Forest disease in Thirthahalli, Karnataka, India, 2014. International Journal of Infectious Diseases

2. The sentence that KFD vaccine has good efficacy is debatable.

Reply: we have omitted the words good efficacy and added a line No. 87-88 “There is limited availability of information on KFD vaccine for human use in India.”

3. The manuscript describes the findings of coverage / barriers to vaccination initiated in 2015. I am not sure if these findings are still valid after 4 years of the outbreak. The coverage might have been improved over the last three years. The barriers identified might no longer be valid now?

Reply: This study describes the vaccination coverage since 2015 to 2018. Moreover, many villages which were affected during 2015 and 2016 are continued to report KFD cases in subsequent years also. Hence we feel the reasons are still valid. Also the overall vaccination coverage from the year 2015-2018 for each of the 40 affected villages was calculated shows that 32 villages had vaccination coverage of less than 50%. Though the vaccination coverage is estimated from 2015 to 2018 the qualitative inquiries are conducted during 2019. Hence, we feel it would reflect more on the contemporary issues.

4. The vaccination strategy

Reply: Thanks for the comment. To make it clear we have added the paragraph on vaccination strategy and schedule in the revised manuscript (line 139-141)

Methods

5. Authors mention that vaccination was conducted in multiple places (CHC, campaigns etc). How did you ensure that persons who came for the second/booster dose of vaccine, in fact had taken earlier doses?

Reply: The line list of patient names were maintained in registers at CHC valpoi and these registers were carried by the ANMs when they used to go for the community level vaccination. Also a doctor used to examine each patient prior to vaccination along with the history of previous vaccination against KFD.

6. Do you think that counselling about the next dose of vaccine could have improved your coverage of dose-2. In UIP vaccines, counselling by health workers for the next dose of vaccine has been found to be an important determinant of complete vaccination (Ref)

Reply: Counseling by health workers as well as the doctor was done at the first dose. The vaccinees were given a vaccination card which also had a mention of date for the follow up doses.

7. Do you think 2 FGDs are sufficient to capture views about vaccination?

Reply: Two FGDs conducted among the community members of affected villages actually went on for an hour. Hence, processing the transcript of this two FGDs were in fact rich and voluminous. Apart from this, we also had Key informant interviews with several key stakeholders. Transcripts of these individual interviews and FGDs had several overlapping issues. Hence, we felt conducting additional FGDs would be redundant and may not lead to emergence of any new themes.

Results

8. Do you think 2 FGDs are sufficient to capture views about vaccination of 40 villages?

Reply: Thanks for the comments. As we shared for the previous comment, In these villages the two FGDs conducted among the community members of affected villages actually went on for an hour. Hence, processing the transcript of this two FGDs were in fact rich and voluminous. Apart from this, we also had Key informant interviews with several key stakeholders. Transcripts of these individual interviews and FGDs had several overlapping issues. Hence, we felt conducting additional FGDs would be redundant and may not lead to emergence of any new themes.

Moreover out of these 40 villages majority belong to same taluka. Hence we feel the people characteristics, occupation, cultural context and perceptions also would reflect the same.

9. 40 villages targeted for vaccination had at least one case. However, the vaccination strategy followed in Karnataka is villages within 5 km radius, which will include villages without any case as well. This appears not the case in Goa.

Reply: In Goa the villages with cases were targeted first followed by few villages in the surrounding. The concept of vaccinating 5kms radius was not strictly followed.

We hope the following lines from the manuscript will give better understanding on the stated issue.

“This study estimates the vaccine coverage in villages where there is occurrence of at least one case in four years. However, there could be villages which had vaccination campaigns based on the policy of being located within 5 kilometres of the affected village or reported monkey deaths from the forest department. These villages were not included in the overall estimation of vaccine coverage, although they were limited in number i.e. 6 villages only. We feel that inclusion of those villages might have lowered the vaccine uptake as we expect less than anticipated response in villages where no case of KFD has been detected.” Line No 375-382.

10. You mention that these villages were targeted for vaccination during 2015-18. But apparently the coverage was reported for 2016 only. If you say that the coverage data is for 2015-18, it would also be appropriate to provide data by year.

Reply: In the year 2015 when the outbreak just started only 499 (300 1^st^ dose and 199 2^nd^ dose) were given that too only at community Health Centre Valpoi for the people from Pali village. Subsequently from March 2016 the vaccination campaigns have started when the vaccine stock was in sufficient supply.

11. Your numbers do not match – you mention in the text that 16, 141 vaccinations were done whereas in the flow chart 16, 015 had received any dose.

Reply: The reason for the difference is Fig 1 has shown up to 1^st^ booster dose. 126 people had received the 2^nd^ yearly booster i.e. a 4^th^ dose. For clarity, we have made a mention of this in line no 261 in the revised manuscript.

12. Fig 2 shows >40 villages, many with zero cases. But in the text you mention only 40 villages with at least 1 case were targeted for vaccination and table-2 gives coverage of these 40 villages. Would not it be appropriate to include these 40 villages in the map

Reply: Thanks for the comment. Majority of the villages were not targeted for vaccination hence these were not highlighted.

It is logical to restrict these 40 villages for mapping. However, to show the clustering of cases and vaccination coverage in the geographical context of Goa we want to include the whole of Goa district shape files in the map. By this way, it could also give understanding of spread from adjoining state as well.

13. In the linear regression, consider including incidence of KFD as an independent variable, rather than taking number of cases and population separately.

Reply: Since the vaccination strategy was shifted from Community Health Centres to Primary health centres there was a great challenge in the mobilization of ANMs to these out-reach campaign. Qualitative interviews also reflected human work force constraints and work load in these vaccination campaigns were also a deterrent factor. Work load is determined by number of eligible people to be vaccinated in that village. Hence we want to retain the target population as the separate variable instead of incidence of KFD cases.

14. You have presented the unadjusted results of linear regression. Consider giving adjusted results by including all variables in the model.

Reply: Thanks for the comments. As per the suggestion we have done adjusted linear regression and reported the co-efficient for adjusted model in the revised manuscript.

| Factor | Correlation | Beta coefficient | P value |
| --- | --- | --- | --- |
| Cumulative number of confirmed KFD cases from 2015-18 | 0.17 | 0.34 | 0.3 |
| Number of targeted population to be vaccinated in a village | -0.07 | -0.003 | 0.4 |
| Distance to the nearest CHC | 0.14 | 0.63 | 0.8 |
| Time taken to reach the nearest CHC | 0.13 | -0.24 | 0.9 |

Dependent Variable: Vaccination coverage for first dose KFD

Independent variables: Cumulative number of KFD cases, Number of targeted population, Distance and time to travel

15. Was the vaccine coverage different by age groups?

Reply: Age wise data was not analyzed. It would not be possible to collect the age wise data from the vaccination registers now as we would have to take permission to access the registers which are maintained at the CHC/PHCs which would take a long time.

16. This was an ecological study and this could be mentioned as a limitation while commenting about the associations.

Reply: Thanks for the comments. Indeed the ecological observations are the major limitations of this study. We have mentioned this under limitation section in line number 402-404.
